# Effect of Processing Methods on Antinutritional Factors (Oxalate, Phytate, and Tannin) and Their Interaction with Minerals (Calcium, Iron, and Zinc) in Red, White, and Black Kidney Beans

**DOI:** 10.1155/2023/6762027

**Published:** 2023-10-18

**Authors:** Serkalem Abera, Weldegebriel Yohannes, Bhagwan Singh Chandravanshi

**Affiliations:** Department of Chemistry, College of Natural and Computational Sciences, Addis Ababa University, P. O. Box 1176, Addis Ababa, Ethiopia

## Abstract

The purpose of this study was to assess how different processing techniques affected mineral compositions, antinutritional factors, and their interactions in red, white, and black kidney beans consumed in Ethiopia. Mineral contents were found to be 41–44, 58–78, and 112–126 mg Ca/100 g in the raw, soaked, and cooked samples, respectively. Iron content in the raw, soaked and cooked samples were found to be 2.77–2.97, 1.94–2.20 and 2.87–3.28 mg Fe/100 g, respectively, showing 26–30% loss on soaking followed by 33–48% increase on cooking. While Zn content in the raw, soaked and cooked samples were found to be 2.47–3.26, 3.34–4.68 and 2.83–3.31 mg Zn/100 g, respectively, showing 35–43% increase on soaking followed by 15–29% decrease on cooking. In the case of antinutrients, both treatments showed incredible decrements. Phytate in the raw samples was 178-179 mg/100 g and showed a 12–16% decrement on soaking and a 37-38% decrement up on cooking, oxalate was 1.5–1.8 mg/100 g in the raw samples and showed a 4.4–13% decrement during treatments, and tannin in the raw samples was 102–160 mg/100 g and showed a 23–30% decrement on soaking, followed by 21–41% during cooking. Phytate : Ca and oxalate : Ca molar ratios in soaked and cooked samples were within the critical values in the raw samples. In contrast, phytate : Zn and Ca × phytate : Zn in all treatments were found to be within the critical value, confirming the good bioavailability of zinc in all the samples, while phytate : Fe was found over the critical value, showing its poor availability.

## 1. Introduction

Kidney bean is one of the main food and income crops in Ethiopia. It is significant for the country's economy and has historically ensured food security in Ethiopia [1]. It ranks third among grain export in Ethiopia, contributing roughly 9.5% of all agricultural exports' total value. In many regions of Ethiopia, it is consumed as the primary traditional cuisine. It is mostly produced in Oromia, South Nations, Nationalities, and Peoples (SNNP), and Amhara regions of Ethiopia, which have respective area coverages of 146,452.41 hectare (ha) (41%), 117,969.97 ha (33%), and 81,235.07 ha (22.74%). It is primarily grown in Ethiopia's eastern, southern, southwestern, and rift valley regions. Remaining 3.25% is produced in other regions of Ethiopia [2]. The southern part of the country, Sidama region, and Gamo Gofa zones produce red and speckled types mainly for home consumption. While in the eastern part, mainly Hararghe highlands, people preferred mostly speckled and white beans [2].

Kidney bean, *Phaseolus vulgaris*, green bean or common bean, is an annual, climbing plant in *Fabaceae* (legume or bean family) in the genus *Phaseolus* L. that sourced in Central and South America and is now cultivated in many parts of the world [3]. Depending on the variety, they may tolerate a wide range of environmental conditions from sea level to about 3000 meters above sea level (m.a.s.l.). However, due to poor pod set brought on by high temperatures, it does not produce fruit below 600 meters [3]. Some of the well-known varieties of kidney beans include red, white, and black ones. These beans are an excellent source of pertinent nutrients and contain a lot of protein, important minerals, and carbohydrates. However, certain antinutritional factors (phytate, oxalate, tannin, and lectin) present in them have an impact on how biologically nutrients are utilized [3].

Minerals are necessary for all living things to be healthy. It is vital to remember that minerals play a combined function in human nutrition and health, just like proteins, carbs, and fats do [4]. One or more of the body's functions are impaired by a mineral shortage. Due to high phytic acid concentration in diets, Zn and Fe are two of the micronutrients that are most frequently deficient in developing nations [4]. The bioavailability of plant-based micronutrients such as iron and zinc is generally influenced more by dietary variables than by macronutrients [4]. The balance between variables that either impede or increase nutrient absorption in the entire diet determines the overall influence on nutrient bioavailability [5].

Phytate is a natural substance known to be antinutritional components in legumes and regarded as a primary storage compound for phosphorus [6]. Essential mineral nutrients are bound by negatively charged phosphate groups in phytic acid, which prevents the body from absorbing them [7]. Phytate particularly affects Zn and Fe, as their deficits have been linked to excessive phytate intake [4, 7].

Oxalate salts are insoluble salts that are naturally found in both the human body and plants. They can exist in the form of soluble salts of potassium and sodium, soluble salts of calcium, magnesium, and iron, or a combination of the two [8]. While soluble oxalate affects the body by blocking the body's ability to absorb dietary calcium and other minerals by building a potent chelate with them, insoluble oxalate is eliminated in urine [9]. The kidney builds up insoluble calcium oxalate in crystal form, generating kidney stones that lead to renal failure. Although water-soluble minerals were also leached out at the same time as the oxalate level was reduced, cooking may produce obvious skin rupture and speed up the leakage of soluble oxalate into cooking water [10].

Tannin is a polyphenolic compound that prevents the body from utilizing vitamins, proteins, and minerals; it is seen to be nutritionally undesirable. It has the capacity to interact with proteins through covalent and hydrogen bonding interactions, which causes proteins to precipitate [11]. They are made up of a remarkably varied assortment of oligomers and polymers [12]. Fortunately, tannins are soluble in water, with the exception of some structures with higher molecular weights.

Despite many advantages that plant-based diets offer, they are less nutrient-dense when ingested improperly [13]. A side effect of eating too much kidney beans may include bloating or gas [14]. However, traditional household practices such as soaking and cooking can decrease the antinutritional content significantly.

The review of the literature reveals that studies on the nutritional and antinutritional composition of red kidney beans have been published from Nigeria, Pakistan, and India [4]. On the other hand, few in-depth studies have been conducted on Ethiopian-originated kidney beans. People are consuming food cooked just after soaking it for a great deal of time (5–10 hours), but there is no guarantee of the food's quality. Some minerals may not be available in sufficient quantities or may not be processed in the best way possible, preventing people from obtaining all resources found in these plants. This study's objectives were to determine how different processing techniques affected antinutrients phytate, oxalate, and tannin as well as to evaluate minerals calcium, zinc, and iron in samples of red, white, and black kidney beans with respect to their respective molar ratios to gauge the body's mineral absorption. The study significantly advanced our knowledge of the levels of antinutritional compounds such as oxalate, phytate, and tannin contained in red, white, and black kidney beans, as well as how these compounds interact with minerals such as calcium, iron, and zinc to affect health and nutrition.

## 2. Materials and Methods

### 2.1. Instruments

An atomic absorption spectrophotometer (AA-6800 AAS Shimadzu, Japan), a UV-Vis spectrophotometer (CECIL, CE 1021, 1000 series, UK), a mechanical shaker (Optima OS-762 Shaker for Incubator 30–300 rpm Load Max 3.0 kg), a vortex mixer (Electro Scientific Industries SI-0286 Vortex Mixer (GENIE2) 3220 rpm/3350 rpm), and a centrifuge (DYNAC II centrifuge, Clay Adams, division of Becton Dickinson and Company, USA) were used.

### 2.2. Chemicals

All solutions were made using chemicals suitable for analytical grade reagents. HClO_4_ (70% (Riedel-de Haën, Germany), HCl (37% Riedel-de Haën, Sigma-Aldrich Chemicals GmbH, Germany), HNO_3_ (about 69% LR, Eurostar Scientific Ltd., UK), sulfosalicylic acid (Merck, Germany), FeCl_3_.6H_2_O (iron(III) chloride crystals, Merck, Germany), sodium phytate salt (phytic acid dodeca sodium salt hydrate water 10–15% product, Aldrich, USA), 3 M H_2_SO_4_ (98%, (Riedel-de Haën, Germany), 0.1 M KMnO_4_ (min. 99%, Alpha Chemika) solution that was preheated for 1 h in the water bath at 90°C, methanol (30%), and vanillin-HCl solution were used, standard metal ion solutions (1000 mg/L) were purchased from AAS Company Vorna Valley (AAS standard, Germany), and freshly prepared deionized water was used in all experiments.

### 2.3. Sample Collection

Red, white, and black kidney bean samples were collected from Sidama (Bansa Daye), Amhara (Bure, Gojam), and Oromia (Arsi Negele) regions of Ethiopia. They were transported to the laboratory, cleaned, dried, and stored in hermetically sealed polyethylene bags for future processing.

### 2.4. Sample Preparation

About 1 kg of red, white, and black kidney beans was manually washed, dried in open air conditions, and divided into two groups (raw and processed). The beans in the processed group were further divided into two equal groups just after soaking for overnight (16 h), and soaking water was drained off. For cooking, half of the soaked samples were cooked in distilled water at 100°C for 60 ± 5 min and allowed to air-dry (at room temperature for 72 h) according to the method used by Ihemeje et al. [15] with little modification of soaking and cooking time. The dried samples (raw, soaked, and cooked) were ground to fine powder using a laboratory electrical grinder and sieved using a sieve of 0.425 mm mesh size, packed into airtight-sealed polyethylene bags, and left for later analysis [15]. The flours of the kidney bean samples were subsequently analyzed for their minerals (calcium, zinc, and iron) and antinutritional factors (phytates, oxalates, and tannins).

### 2.5. Soaking

The red, white, and black kidney beans were all individually steeped in distilled water for 16 hours at room temperature in the dark with little modification of soaking hours [15]. After that, the soaking water was drained out. The seeds were divided into two halves, with one of them being air-dried for 72 hours to ensure full drying. Samples were ground to pass through a 30-mesh screen. The ground samples were kept in airtight bottles and stored at 4°C for subsequent analysis [15].

### 2.6. Cooking

The presoaked seeds of red, white, and black kidney beans were cooked on a hot plate (about 60 ± 5 min) until they were readily crushed with the thumb and index fingers. The cooking liquid and seeds were separated by filtration, and the cooked seeds were dried in the same manner as after the soaking phase [15].

Each of the three sets of samples was analyzed in triplicates for their oxalate, phytate, tannin, and minerals (Ca, Fe, and Zn). An analysis of samples was carried out according to the available standard/published methods depending on the availability of lab requirements. UV-Vis spectrophotometry (for phytate and tannin), atomic absorption spectrometry (for calcium, iron, and zinc), and the KMnO_4_ titrimetry method (for oxalate) were used, respectively [15–17].

### 2.7. Digestion for Mineral Determination

Exactly, 0.5 g of homogenized and powdered red, white, and black kidney beans was transferred separately into a 250 mL round-bottomed flask and connected to a reflux condenser. 4 mL of 69% nitric acid (HNO_3_) and 1 mL of 70% perchloric acid (HClO_4_) (4 : 1 ratio) at a temperature of 300°C were added to the flask. The mixture was heated for 3 h over a hot plate until the solution was clear and colorless. Then, the solution was allowed to cool for 20 min before the condenser was dismantled and 5 min after dismantling the condenser. Thereafter, 5 mL of deionized water was added, and the solution was filtered through Whatman No. 41 filter paper into a 25 mL volumetric flask. This procedure was followed according to the method developed by Ayele et al. [4] and Maki et al. [16] with some modification in the type and volume of reagents. The filter paper was washed thoroughly, and washing was collected in the flask. To decrease interferences on calcium from phosphates, sulfates, or anticoagulants such as oxalates, citrates, and heparin, 2.5 mL of 10% lanthanum chloride solution was added to the flask since calcium needs to be measured [4]. Finally, it was diluted to the mark (25 mL) with deionized water. The blank was prepared by taking the same amount of reagent through all steps.

### 2.8. Determination of Phytate, Oxalate, and Tannin

Oxalate was determined by the AOAC 2005 method [17], tannin was determined by the vanillin-HCl assay method using a UV spectrophotometer [18], and the phytate content was determined by the method described by Latta and Eskin [19] and later modified by Vaintraub and Lapteva [20].

The molar ratio was calculated by dividing the mole of antinutrient to the mole of minerals [4, 21]. To assess the impact of high levels of phytate and oxalate on the bioavailability of dietary minerals (zinc, calcium, and iron), the molar ratios of phytate to calcium, phytate to iron, phytate to zinc, oxalate to calcium, and phytate to calcium/zinc computed as the ratios are the better indicators of bioavailability than the amounts of mineral and phytic acid in the diet [16].

### 2.9. Recovery (Validation and Accuracy)

Accuracy of an analytical method is the degree of agreement of test results generated by the method to the standard true value which is estimated by spiking the sample with a known amount of a standard of 1000 mg/L stock solution [22]. A known concentration of 40% of the analyzed minerals (calcium, iron, and zinc) and antinutrients (phytate, oxalate, and tannin) was added to 0.5 g of powdered kidney bean samples in three treatments (raw, soaked, and cooked). The spiked samples were digested and analyzed at optimum conditions as the method to be validated, and the outcomes were contrasted with the anticipated rise in the parameter to be studied in comparison to the raw data [4].

The limit of detection (LOD) is the lowest analyte concentration that produces response detectable but not necessarily quantifiable above the noise level of the system. It is calculated as LOD = 3 × SD_blank_ [22]. Low LOD indicates the presence of trace amounts of metals of interest in the sample that can be detected by the given method. The limit of quantification (LOQ) is the smallest quantity of analyte that can be measured with acceptable accuracy and precision, which is calculated as LOQ = 10 × SD_blank_ [22]. The linear range and precision were tested for the characteristics for the determination of minerals and antinutrients [22]. The wavelength, limit of detection (LOD), limit of quantification (LOQ), correlation coefficient (*R*^2^), and calibration curve equation for mineral determination in kidney bean samples are given in Table S1. As can be seen from the table, the wavelength used for the determination of Ca, Zn, and Fe is 422, 213, and 248 nm, respectively. LOD and LOQ are low enough to determine the trace levels of the three metals of interest. The correlation coefficients (*R*^2^) of the calibration curves are greater than 0.999 for all the three metals which show an excellent relationship between the absorbance and concentration of the three metals.

### 2.10. Statistical Analysis

Mineral composition and antinutritional factors of the raw and processed samples of three different types of kidney bean were statistically compared using the analysis of variance (ANOVA) and least significant difference (LSD). The statistical package used was SPSS version 25. Significant differences were determined at a *p*  <  0.05 level [17]. All the results for minerals (calcium, iron, and zinc) and antinutritional factors (oxalate, phytate, and tannin) were reported as the mean value with their respective standard deviations. The results were reported as mg analyte/100 g of raw, soaked, and cooked samples for consistency and comparison.

## 3. Results and Discussion

### 3.1. Calibration Curve for Minerals and Antinutrients

By serially diluting a 1000 mg/L standard stock solution with deionized water and taking precise measurements of the standard solutions and reagents, respectively, the intermediate and working standards solutions of each mineral (zinc, iron, and calcium) and antinutrients (phytate, oxalate, and tannin) were prepared [15]. The calibration curve was obtained by running a series of prepared working standards for all minerals and antinutrients. The correlation coefficient varies from 0.9974 to 0.9996 for minerals and 0.9974 to 0.9975 for antinutrients, which shows a very good linearity of the curves. The calibration curves are given as supporting information (Figures S1 and S2). The correlation coefficients (*R*^2^) of the calibration curves of three metals clearly show that there is a good relationship between the absorbance and concentration of the three metals (Figure S1). Similarly, the correlation coefficients (*R*^2^) of the calibration curves of two antinutrients clearly show that there is a good correlation between absorbance and the two antinutrients (Figure S2).

### 3.2. Minerals

**Figure 1:**
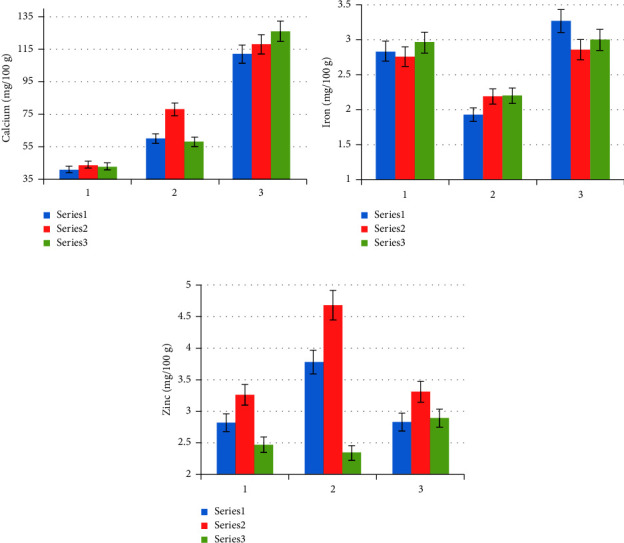
Mineral contents (a) Ca, (b) Fe, and (c) Zn (mg/100 g) of raw (blue series 1), soaked (red series 2), and cooked (green series 3) of red (1), white (2), and black (3) kidney bean samples as the mean ± standard deviation of the triplicate (*n* = 3).

The mineral contents of the raw, soaked, and cooked red, white, and black kidney bean samples are shown in Figure 1.

#### 3.2.1. Calcium

Calcium content showed a significant difference (*p*  <  0.05) in both processing conditions (soaking and cooking) for all kinds of kidney bean samples (red, white, and black). The effect of soaking and cooking on the calcium content showed an increment from 41–44 mg/100 g for the raw sample to 58–78 mg/100 g and 112–126 mg/100 g for the soaked and cooked samples, respectively. This implies that the processing method shows a significant increment of calcium from 41.46 to 43.59% during soaking and 38.19 to 48.21% upon cooking, which is similar to the results reported by Akin-Idowu et al. [19] that explained an increment of calcium on soaking followed by cooking. The reason for a significant increment of calcium content during soaking is most probably due to loss of soluble minerals and other components in the soaking water. The Ca-oxalate complex is insoluble in water and hence remains in kidney beans, resulting in the increase in the calcium content in the kidney beans after soaking.

#### 3.2.2. Iron

The iron content shows a significant difference (*p*  <  0.05) between the raw, soaked, and cooked conditions in all the samples (red, white, and black kidney beans). Iron content in the raw, soaked and cooked samples were found to be 2.77–2.97, 1.94–2.20 and 2.87–3.28 mg Fe/100 g, respectively, showing 26–30% loss on soaking followed by 33–48% increase on cooking. This might be because of leaching of iron. Upon cooking, the content of iron increased from 1.94 to 3.28 mg/100 g, 2.20 to 2.87 mg/100 g, and 2.21 to 3.01 mg/100 g for the red, white, and black kidney beans, respectively, which means it showed a 32.62–47.93% increment during cooking, which is similar to the result reported by Omoruyi et al. [23]. This might be due to the releasing of iron from antinutritional factors, mainly phytate.

#### 3.2.3. Zinc

The levels of zinc in all samples showed increments during soaking. The results obtained were 2.82–3.78 mg/100 g, 3.26–4.68 mg/100 g, and 2.47–3.34 mg/100 g for the red, white, and black kidney bean samples, respectively, which means it showed a 26.04–43.55% increment during soaking. The result is consistent with the one reported by Akin-Idowu et al. [19]. But loss of zinc was observed after cooking. The results were 3.78−2.83, 4.68−3.31, and 3.34−2.89 mg/100 g for the red, white, and black kidney bean samples, respectively, which means a 15.3–29.27% decrement was observed during cooking that might be due to leaching with the cooking water.

### 3.3. Antinutrients

**Figure 2:**
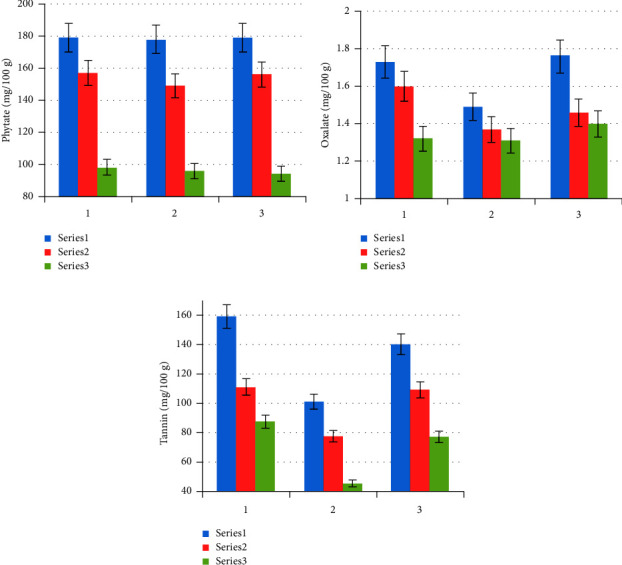
Antinutritional factor contents (a) phytate, (b) oxalate, and (c) tannin (mg/100 g) of raw (blue series 1), soaked (red series 2), and cooked (green series 3) of red (1), white (2), and black (3) kidney bean samples as the mean ± standard deviation of the triplicate (*n* = 3).

Antinutritional contents of red, white, and black kidney bean samples in raw, soaked, and cooked conditions are shown in Figure 2.

#### 3.3.1. Phytate

From all the antinutritional factors, phytic acid is considered as one of the main problems for human health and nutrition [15]. Soaking was previously reported to reduce phytates [16] due to leaching of phytate ions into the soaking water. The same thing happened in our research for both treatments. Furthermore, water consumption activates the bean's phytase enzymes, which decrease and breakdown phytates [10].

In our study, the effect of soaking and cooking showed a significant difference at *p*  <  0.05, and the loss of phytate during both treatments is 37.3%, 35.9%, and 61.7 39.6%, respectively, for the red, white, and black kidney bean samples, which is higher than in the result reported by Bhandari and Kawabata [24]. They reported the average loss of phytates during cooking to be 20% which coincides with the result reported by Ihemeje et al. [15] that reported a 32.8–44.7% decrement of phytate upon cooking. In this study, the highest amount of phytate is observed in red kidney bean, which is 179, 157, and 98.2 mg/100 g for raw, soaked, and cooked beans, respectively. Phytate mainly reduces the bioavailability of dietary zinc by forming insoluble mineral chelates [22].

#### 3.3.2. Oxalate

Oxalate is reported to have an effect comparable to that of phytate [22]. Oxalate mainly binds calcium and makes it unavailable for absorption by the body, causing the formation of kidney stones [25]. It significantly affects the availability of calcium only when the ratio of oxalate: Ca is greater than one [26].

In our findings, almost the same amounts of oxalate are found in all types of kidney beans, and a significant loss of oxalate is not expected if the soaking and cooking water is not discarded after all [27] because the higher percentage of oxalate reduction during cooking may also be due to its solubility in boiling water.

#### 3.3.3. Tannin

Tannin shows a significant difference (*p*  <  0.05) in both treatments. The highest amount of tannin in all treatments is observed in red kidney bean, which is 160, 112, and 88.8 mg/100 g for raw, soaked, and cooked beans, respectively. The values were very small as compared to 3833–4533 mg/100 g as reported by Ruchi and Sheet [28] on red kidney beans (raw and processed). The reduction in the tannin content may be due to leaching of polyphenols into the soaking water [29, 30].

### 3.4. Molar Ratios and Bioavailability of Minerals

Bioavailability is the percentage of a mineral's total amount that may be absorbed in a form that is metabolically active [31]. The calculated values of the molar ratios were also compared with the reported critical values for these ratios [31]. Phytate : Ca, phytate : Fe, phytate : Zn, and Ca × phytate/Zn molar ratios of red, white, and black kidney bean samples under three processing conditions are given in Table 1.

#### 3.4.1. Phytate : Ca

The phytate : Ca molar ratios in two treatments (soaked and cooked) are less than 0.24, which shows the good bioavailability of calcium [25]. But they vary from 0.24 to 0.26 for raw samples which will have less bioavailable calcium for the body because they are trapped by antinutrients mainly by oxalate and phytate and released during the treatments [19].

#### 3.4.2. Phytate : Fe

The phytate : Fe molar ratios are greater than one in both for all kinds of samples, which is a sign of poor iron bioavailability [32]. Processing methods showed a bit increment on iron content, especially cooking, but do not make it sufficiently available.

#### 3.4.3. Phytate : Zn

The phytate : Zn molar ratios of both processing conditions are less than 15 and indicate good bioavailability of zinc [25, 32]. The bioavailability of zinc is reduced by phytate [32]. Hence, the phytate : Zn molar ratio is considered a better signal of zinc bioavailability than total dietary phytate levels alone [25]. For the good bioavailability of zinc, at least phytate : Zn molar ratios need to be within a range of 10–15 [32].

#### 3.4.4. Phytate × Calcium to Zinc (Ca × Phytate/Zn)

The molar ratios of Ca × phytate/Zn of samples range from 5.94 to 10.0. The potent effect of calcium on zinc absorption in the presence of high phytate intakes has led to the suggestion that the phytate × Ca/Zn millimolar ratio may be a better index of zinc bioavailability than the phytate : Zn molar ratio alone [26]. High calcium levels in foods can promote the phytate-induced decrease in zinc bioavailability when the Ca × phytate/Zn molar ratio exceeds 0.5 mol/kg [32]. In this study, the values of all the samples were within the critical molar ratios of Ca × phytate/Zn, which indicates the bioavailability of zinc is not affected by the kinetic synergism between calcium and zinc.

#### 3.4.5. Oxalate to Calcium Ratio (Oxalate : Ca)

To know the progress of availability of minerals from reduction of oxalate content after soaking followed by cooking, the oxalate to calcium (oxalate : Ca) molar ratio is calculated, and it is all within the critical values (<1), which implies that oxalate cannot have any adverse effects on bioavailability of dietary calcium in all types of samples just after proper treatments. The results are given in Table 2.

### 3.5. Comparison of Mineral (Ca, Fe, and Zn) and Antinutrient (Phytate, Oxalate, and Tannin) Contents of Ethiopian Red, White, and Black Kidney Beans with Those of Other Reported Literature

It has been observed that a food crop's mineral and antinutritional compositions are directly correlated with its genetic background, geographic origin, and soil characteristics [29]. Therefore, it is crucial to see our results of Ethiopian kidney beans compared with those of other reported literature from different parts of the world. The comparison between this study (kidney beans from Ethiopia) and other reported literature (kidney beans from India, Nigeria, Kenya, Pakistan, and West Africa) is given in Tables 3 and 4.

The general trend in the calcium content in the raw, soaked, and cooked kidney beans found in the present study is similar to that reported from India and Nigeria. However, there is a wide variation in the values of calcium contents in the kidney beans from the three countries (Ethiopia 41–112, India 58–112, and Nigeria 28–32.5 mg/100 g). These values are different from that reported from Pakistan (54.2–56.3), which shows no change in the calcium content upon soaking and cooking. The general trend in the iron content in the raw, soaked, and cooked kidney beans found in the present study is similar to that reported from all the other countries. Although there is a variation in the value of iron contents among the five countries, the iron content of Ethiopian kidney beans (1.94–3.28 mg/100 g) is lower that from Pakistan (7.4–11.5 mg/100 g) and Kenya (4.9–6.8 mg/100 g) but higher than that from India (0.89–1.25 mg/100 g) and Nigeria (1.2–1.9 mg/100 g). The zinc content in the kidney beans from Ethiopia (2.82–3.78 mg/100 g) and other countries (Pakistan 2.3–3.0, India 3.0–6.4, and Kenya 1.8–3.7 mg/100 g) does not show any clear trend. The zinc content is not reported in the kidney beans from Nigeria.

The general trend in the phytate content in the raw, soaked, and cooked kidney beans found in the present study is similar to that reported from Nigeria, Kenya, and West Africa. While kidney beans from Pakistan showed a very high phytate content (610–630 mg/100 g) and did not show any effect on the phytate content upon soaking and cooking. The phytate content of Ethiopian kidney beans (179−98.2 mg/100 g) is lower than that reported from Nigeria (320−177 mg/100 g), Kenya (207−178 mg/100 g), and West Africa (341−194 mg/100 g). The general trend in the oxalate content in the raw, soaked, and cooked kidney beans found in the present study is similar to that reported from Nigeria and West Africa. However, the oxalate content of Ethiopian kidney beans (1.79−1.32 mg/100 g) is much lower than that from Nigeria (18.3−10.0 mg/100 g) and West Africa (115−94 mg/100 g). The oxalate content in kidney beans from Pakistan and Kenya is not reported. The general trend in the tannin content in the raw, soaked, and cooked kidney beans found in the present study is similar to that reported from Nigeria, Kenya, and West Africa but different from that of Pakistan. The tannin content of kidney beans from Ethiopia (160−80.4 mg/100 g) is higher than that from Nigeria (78−56 mg/100 g) and similar to that from Kenya (163−63 mg/100 g) and West Africa (112−59 mg/100 g).

## 4. Conclusion

This study gives information on the mineral contents (Zn, Fe, and Ca) present in samples of red, white, and black kidney beans of Ethiopian origin and the effects of antinutritional factors (phytate, oxalate, and tannin) found in them under two treatments. In addition, the relative bioavailability of the minerals is assessed by calculating molar ratios of antinutrients to the minerals, and the results are compared with the critical values to confirm the bioavailability of minerals. The results obtained from the study showed that both treatments (soaking and cooking) significantly reduce antinutritional factors and increase the bioavailability of the minerals. Processing methods resulted in a significant increment of calcium (41.46–43.59%) during soaking and 38.19–48.21% upon cooking. Iron showed a decrease just after soaking (26–30%), followed by a 33–48% increment upon cooking. While an increment of zinc was observed upon soaking, it resulted in a 15–29% decrease during cooking. In contrast to minerals, both treatments displayed an astounding decrease in antinutritional factors. Phytate showed a 12–16% decrease upon soaking, followed by a 37–38% decrease upon cooking. Oxalate resulted in a 4.4–13% decrease in both treatments. Tannin showed a 23–30% decrease upon soaking, followed by a 21–41% decrease during cooking. Phytate : Ca and oxalate : Ca molar ratios in soaked and cooked samples were within the threshold ranges in the raw samples. The phytate : Zn and Ca × phytate : Zn molar ratios in all treatments were found to be within the critical value, proving the good bioavailability of zinc in all samples, while phytate : Fe was shown to be above the critical value, demonstrating its poor availability. All kinds of kidney beans are used as a source of calcium and zinc, while the phytate : Fe molar ratio indicates that iron from all kinds of kidney beans is not bioavailable. The results obtained from the study showed that both treatments (soaking and cooking) significantly reduce antinutritional factors and increase the bioavailability of the minerals. The measured antinutrient to mineral ratio also demonstrated the effectiveness of the strategies for lowering antinutrients that might improve the nutritional characteristics of kidney beans. The results found showed that there is no significant difference between the three types of kidney beans in terms of nutritional quality (minerals concentrations) and antinutritional factor contents.

## Figures and Tables

**Table 1:** Phy : Ca, Phy : Fe, Phy : Zn, and Ca × Phy/Zn molar ratios of red, white, and black kidney bean samples under three processing conditions.

Samples	Phytate (mmol/100 g)	Ca (mmol/100 g)	Zn (mmol/100 g)	Fe (mmol/100 g)	Phytate : Ca molar ratio	Phytate : Zn molar ratio	Phytate : Fe molar ratio	(Ca × phytate/Zn)
RR	0.27	1.02	0.043	0.051	0.26	6.30	5.31	6.40
SR	0.24	1.5	0.058	0.035	0.16	4.10	6.77	6.21
CR	0.15	2.8	0.044	0.059	0.051	3.36	2.51	9.54
RW	0.27	1.1	0.050	0.049	0.24	5.4	5.51	5.94
SW	0.23	1.94	0.072	0.039	0.12	3.14	5.79	6.25
CW	0.15	2.95	0.051	0.051	0.051	2.84	2.84	8.63
RB	0.27	1.07	0.038	0.053	0.25	7.13	5.11	7.63
SB	0.24	1.45	0.051	0.039	0.16	4.63	6.05	6.86
CB	0.14	3.14	0.044	0.054	0.04	3.23	2.63	10
Critical values					<0.24	<15	<1	<0.5 mol/kg

Sample code: RR: raw red; RW: raw white; RB: raw black; SR: soaked red; SW: soaked white; SB: soaked black; CR: cooked red; CW: cooked white; CB: cooked black.

**Table 2:** Oxalate : calcium molar ratios of red, white, and black kidney bean samples.

Samples	Calcium (mol/100 g)	Oxalate (mol/100 g)	Oxalate : Ca molar ratio
RR	1.02	0.019	0.02
SR	1.5	0.017	0.01
CR	2.8	0.014	0.005
RW	1.1	0.016	0.01
SW	1.94	0.015	0.007
CW	2.95	0.014	0.005
RB	1.07	0.019	0.02
SB	1.45	0.016	0.012
CB	3.14	0.015	0.005
Critical values	<1		

Sample code: RR: raw red; RW: raw white; RB: raw black; SR: soaked red; SW: soaked white; SB: soaked black; CR: cooked red; CW: cooked white; CB: cooked black.

**Table 3:** Comparison of minerals (Ca, Zn, and Fe) found in Ethiopian red kidney bean with other reported values mg/100 g.

Countries	Processing conditions	Amount of minerals (mg/100 g)			References
		Calcium	Iron	Zinc	
Ethiopia	Raw	41	2.85	2.82	This study
	Soaked	60	1.94	3.78	
	Cooked	112	3.28	2.83	

Pakistan	Raw	54.9	11.5	2.7	[33]
	Soaked	54.2	7.4	2.3	
	Cooked	56.3	10.5	3.0	

Nigeria	Raw	28	1.5	NR	[15]
	Soaked	30	1.2	NR	
	Cooked	32.5	1.9	NR	

India	Raw	58	0.89	3.0	[34]
	Soaked	NR	NR	NR	
	Cooked	112	1.25	6.4	

Kenya	Raw	NR	6.5	1.8	[32]
	Soaked	NR	4.9	3.7	
	Cooked	NR	6.8	3.2	

NR = not reported.

**Table 4:** Comparison of antinutrients (phytate, oxalate, and tannin) found in Ethiopian red kidney bean with other reported values mg/100 g.

Countries	Processing conditions	Antinutrients (mg/100 g)			References
		Phytate	Oxalate	Tannin	
Ethiopia	Raw	179	1.79	160	This study
	Soaked	157	1.60	112	
	Cooked	98.2	1.32	88.4	

Pakistan	Raw	610	NR	610	[33]
	Soaked	610	NR	630	
	Cooked	630	NR	110	

Nigeria	Raw	320	18.3	78	[15]
	Soaked	215	15.2	87	
	Cooked	177	10.0	56	

Kenya	Raw	207	NR	163	[35]
	Soaked	189	NR	106	
	Cooked	178	NR	63	

West Africa	Raw	341	115	112	[36]
	Soaked	291	109	110	
	Cooked	194	94	59	

NR = not reported.

## Data Availability

The data used to support the findings of this study are included within the article.

## Supplementary Materials

Supplementary Materials Table S1: Wavelength, limit of detection (LOD), limit of quantification (LOQ), correlation coefficient (R^2^), and calibration curve equation for mineral determination in kidney bean samples. Figure S1: calibration curves for determination of (a) Ca, (b) Zn, and (c) Fe. Figure S2: calibration curves for determination of (a) phytate and (b) tannin.
