# Supplementary material for: Effect of Processing Methods on Antinutritional Factors (Oxalate, Phytate, and Tannin) and Their Interaction with Minerals (Calcium, Iron, and Zinc) in Red, White, and Black Kidney Beans
Source: Int J Anal Chem. 2023 Oct 18;2023:6762027. doi: 10.1155/2023/6762027 (PMC10599953; doi:10.1155/2023/6762027)
Supplement: Supplementary Materials — Table S1: Wavelength, limit of detection (LOD), limit of quantification (LOQ), correlation coefficient (R2), and calibration curve equation for mineral determination in kidney bean samples. Figure S1: calibration curves for determination of (a) Ca, (b) Zn, and (c) Fe. Figure S2: calibration curves for determination of (a) phytate and (b) tannin. [file 6762027.f1.docx]

**Supporting Information**

TABLE S1: Wavelength, limit of detection (LOD), limit of quantification (LOQ), correlation coefficient (R^2^) and calibration curve equation for mineral determination in kidney bean samples

| Metals | Wavelength (nm) | LOD  (mg/L) | LOQ  (mg//L) | Correlation coefficient | Calibration curve equation |
| --- | --- | --- | --- | --- | --- |
| Ca | 422 | 0.267 | 2.167 | R^2^ = 0.9987 | y = 0.02x-0.0007 |
| Zn | 213 | 0.073 | 0.175 | R^2^ = 0.9998 | y = 0.4219x-0.0013 |
| Fe | 248 | 0.189 | 1.899 | R^2^ = 0.9993 | y = 0.0546x-0.0121 |

(a) (b)

(c)

FIGURE S1: Calibration curves for determination of (a) Ca, (b) Zn and (c) Fe.

(a) (b)

FIGURE S2: Calibration curves for determination of (a) phytate and (b) tannin.
